# COPD and Gut–Lung Axis: How Microbiota and Host Inflammasome Influence COPD and Related Therapeutics

**DOI:** 10.3389/fmicb.2022.868086

**Published:** 2022-04-01

**Authors:** Ling Qu, Qing Cheng, Yan Wang, Hui Mu, Yunfeng Zhang

**Affiliations:** ^1^Department of Respiratory and Critical Care Medicine, Shanghai Putuo District Liqun Hospital, Shanghai, China; ^2^Department of Science and Education, Shanghai Putuo District Liqun Hospital, Shanghai, China; ^3^Department of Clinical Laboratory, Shanghai Putuo District Liqun Hospital, Shanghai, China

**Keywords:** chronic obstructive pulmonary disease, COPD, gut-lung axis, microbiota, nutrition

## Abstract

The exact pathogenesis of chronic obstructive pulmonary disease (COPD) remains largely unknown. While current management strategies are effective at stabilizing the disease or relief the symptoms, new approaches are required to target underlying disease process and reverse lung function deterioration. Recent research showed that pneumonia bacteria is critical in disease progression and gut microbiome is likely perturbed in COPD, which is usually accompanied by a decreased intestinal microbial diversity and a disturbance in immune system, contributing to a chronic inflammation. The cross-talk between gut microbes and lungs, termed as the “gut-lung axis,” is known to impact immune response and homeostasis in the airway. Although the gut and respiratory microbiota exhibit compositional differences, the gut and lung showed similarities in the origin of epithelia of both gastrointestinal and respiratory tracts, the anatomical structure, and early-life microbial colonization. Evidence showed that respiratory infection might be prevented, or at least dampened by regulating gut microbial ecosystem; thus, a promising yet understudied area of COPD management is nutrition-based preventive strategies. COPD patient is often deficient in nutrient such as antioxidant, vitamins, and fiber intake. However, further larger-scale randomized clinical trials (RCTs) are required to establish the role of these nutrition-based diet in COPD management. In this review, we highlight the important and complex interaction of microbiota and immune response on gut-lung axis. Further research into the modification and improvement of the gut microbiota and new interventions through diet, probiotics, vitamins, and fecal microbiota transplantation is extreme critical to provide new preventive therapies for COPD.

## Introduction

Chronic obstructive pulmonary disease (COPD), which affects over 400 million people globally, is characterized by increasing breathlessness, chronic cough, and sputum production that are associated with irreversible progressive inflammatory condition and major lung destruction with airflow limitation (Raherison and Girodet, 2009). The classification of severity of COPD includes four stages based on spirometry and the degree of worsening airflow limitation from stage I to IV (Wedzicha and Seemungal, 2007; Vestbo et al., 2013). The mechanism of the occurrence and development remains largely unknown (Agusti et al., 2020). Evidence showed that COPD is a polygenic disorder with the involvement of epigenetic components. There are multiple environmental risk factors such as cigarette smoking, bacteria/virus infection, and environmental pollutant exposure that could affects a range of potential lung function trajectories through life. Although there is no cure for COPD, early diagnosis and treatment from a multidisciplinary approach is critical for preventing or slowing the progression of COPD and reducing mortality (Criner et al., 2018).

**Figure 1:**
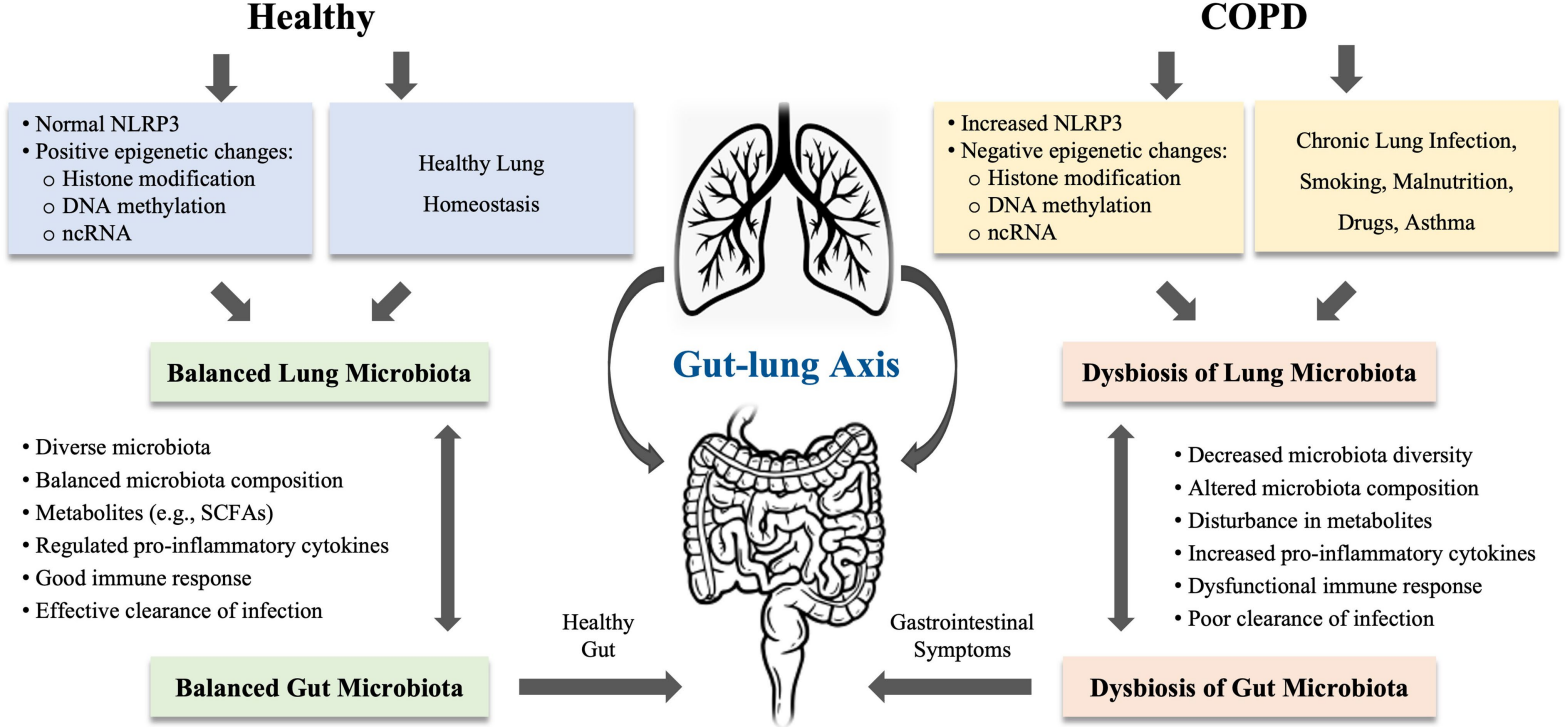
The proposed mechanisms of gut-lung axis involved in chronic obstructive pulmonary disease (COPD). The gut-lung axis concept postulated that disturbance in gut microbiota may have a profound effect on lung disease. The microbiota in gut and lung is closely linked to each other. The microbiota in both organs can be influenced by chronic lung infections, smoking, malnutrition, drugs, and asthma. COPD is highly associated with the dysbiosis in the gut and lung, which can be induced by epigenetic changes or regulated *via* NLRP3 inflammasome. In COPD, disturbance in gut-lung axis leads to decreased microbiota diversity, altered microbiota composition, disturbance in metabolites, increased production of proinflammatory cytokines, and dysfunctional immune response, resulting in poor clearance of infection in the lung or gastrointestinal symptoms or diseases. NLRP3, nucleotide-binding oligomerization domain (NOD)-like receptor (NLR) family, pyrin domain-containing protein; ncRNA, non-coding RNA; and SCFAs, short-chain fatty acids.

Growing evidence revealed that dysbiosis of gut microbiota is considered as an important component in the pathophysiology of COPD (Chotirmall et al., 2017; Shukla et al., 2017; Chunxi et al., 2020). The gut and lungs are anatomically distinct, but both are originated from endosome and abundant bacterial communities (Mjosberg and Rao, 2018). A balanced microbial community in both gut and lungs is important in regulating immune function and overall health of the host. Disturbance in gut microbiota is shown to impact multiple distant organs including the lung. The cross-talk between gut microbes and lung microbes is termed as the “gut-lung axis.” This axis facilitates the passage of endotoxins, microbial metabolites, cytokines, and hormones into the bloodstream connecting the gut and lung. The composition of pneumonia bacteria can be altered by changes in intestinal microbiota and vice versa (Mateer et al., 2015, 2018; Budden et al., 2017, 2019). The gut-lung axis concept postulated that disturbance in gut microbiota may have a profound effect on lung disease (Al Bander et al., 2020). Thus, the bacteria in the intestinal tract can exert either good or bad impact on the lung health (see Figure 1).

Evidence from the gut-lung axis research on respiratory diseases such as asthma, COPD, cystic fibrosis, and respiratory infections suggested that respiratory diseases might be prevented or at least can be ameliorated by regulating gut microbial ecosystem through manipulation of gut microbiota (Zhang D. et al., 2020). Interventions including antibiotics, probiotics, prebiotics, and natural products or diet have shown positive outcomes on balancing gut microbiota and enhancing immune response, but the underlying mechanisms of these therapeutic approaches remain unclear (Zhang D. et al., 2020). COPD patient is often deficient in nutrient such as antioxidant, vitamins, and fiber intake. Nutrition-based preventive strategies might be a promising new interventional strategy for COPD management, especially in a long-term setting (Schols et al., 2014). In addition, further larger-scaled RCTs are required to establish the role of these nutrition-based diet in the management of COPD patients. In this review, we evaluate the role of the gut-lung axis in disease progression and update recent research on the treatment of COPD *via* the gut-lung axis. This review highlights the necessity to develop non-drug interventions as effective adjunctive treatment for COPD.

## Host-Microbial Mutualism in the Gut-Lung Axis

### Gut and Lung Microbiota

The gut microbiota established a close symbiotic relationship with our body that plays a critical role in health maintenance, promoting metabolism of dietary components, vitamin synthesis, preventing pathogen colonization, and regulating immune response (Jandhyala et al., 2015). The gut microbiota is a complex community of microorganisms inhabiting the gastrointestinal tract that in a health individual comprises 150 distinct bacterial species, mainly *Bacteroidetes*, *Firmicutes*, *Proteobacteria*, *Actinobacteria*, and *Verrucomicrobia* (Qin et al., 2010). Besides, fungi have been identified as an integral part of commensal flora, and the diversity of the gut microbiota in healthy subjects is limited to few genera, with a high prevalence of *Saccharomyces cerevisiae*, *Malassezia restricta*, and *Candida albicans* (Nash et al., 2017). Fungi are 100 to 1,000 times larger than bacteria, but less frequent than bacteria sequences. Fungi may compensate the bacterial functions of immune modulation and protect mucosal tissue damage at the physiological state and during gut microbiota disturbances (Jiang et al., 2017). In addition, bacteria can affect the function of fungi (Tso et al., 2018).

While the lung and gut originate from the same embryonic organ in the primitive foregut, the microbiota of lung and gut differ significantly in taxonomic composition, diversity, and function (Girosi et al., 2006). The lung microbiota is composed of transient microorganisms mainly derived from upper respiratory tract (URT) including nostrils, nasal passages, paranasal sinuses, nasopharynx, and oropharynx. While *Bacteroidetes* and *Firmicutes* are the most abundant bacterial phyla in both microbiotas, the lung and gut microbiota are very different at the species level. In the lungs, the genera *Streptococcus* spp., *Veillonella* spp., and *Prevotella* were abundant, whereas *Bacteroides*, *Faecalibacterium*, and *Bifidobacterium* are more prevalent in the gut (Sender et al., 2016). In a disease or dysbiotic state, other organisms such as viruses or fungi are present in the lung (Limon et al., 2017).

### Lung Microbiota in COPD

Lung microbiota plays key roles in the development and progression of COPD. The disease is associated with dysbiosis of the lung microbiota with the overgrowth of pathogenic bacteria, resulting in accelerated decline in lung function. Studies on the lung microbiota of COPD showed reduced lung microbial community diversity compared with those of healthy individuals, with an expansion of *Hemophilus* spp. *Afipia*, *Brevundimonas*, *Curvibacter*, *Moraxella*, *Neisseria*, and *Undibacterium* spp. of the *Proteobacteria* phylum, *Corynebacterium* spp. of the *Actinobacteria* phylum, *Capnocytophaga* spp. of the *Bacteroidetes* phylum, and *Leptolyngbya* spp. of the *Cyanobacteria* phylum (Erb-Downward et al., 2011; Garcia-Nunez et al., 2014; Huang et al., 2014; Millares et al., 2014; Wang et al., 2016). Furthermore, the severity of COPD is associated with less diverse lung microbiota and an expansion of pathogenic microbes (Garcia-Nunez et al., 2014; Wang et al., 2016; Mayhew et al., 2018; Wang Z. et al., 2018). The culture of bronchial secretion from the lower airway of stable COPD patients showed bacteria such as *H. influenza*, *S. pneumoniae*, and *Moraxella catarrhalis*. In contrast, the exacerbated COPD patients present an unstable lung microbiota with an increased relative abundance of *Moraxella* spp. of the *Proteobacteria*, but a decreased abundance of the *Firmicutes* (Rosell et al., 2005; Wang et al., 2016; Sun et al., 2020). Notably, increased *Proteobacteria*: *Firmicutes* ratio suggested that the cause of exacerbation is due to bacterial dysbiosis, whereas eosinophilic exacerbation exhibits a decrease in *Proteobacteria*: *Firmicutes* ratio (Wang et al., 2016). Disturbance in core lung microbiota during acute exacerbation of COPD (AECOPD) leads to the outgrowth of pathogen including *Acinetobacter* spp. and *Klebsiella* spp., indicating that commensal lung microbiota protects the lung from colonization of pathogenic microbes (Sun et al., 2020). Reduced microbial diversity in COPD showed negative correlation with CXCL8, indicating that reduced species diversity might be associated with clearance by neutrophil extracellular traps through invading host epithelial cells. Furthermore, mannose binding lectin (MBL) was shown to interferes with phagocytosis of *H. influenza*. Study by Dicker et al. (2018) showed that COPD patients with MBL deficiency have a more diverse lung microbiota and a lower risk of exacerbation. Additionally, viral infections such as rhinoviruses and influenza virus are detected in around 10%–15% of sputum sample from stable COPD patients in comparison with 30%–60% in severe COPD patients (Mallia et al., 2011; Utokaparch et al., 2014; Tan et al., 2016). Moreover, the mycobiome in the lung microbiota showed an increased relative abundance of *Candida* spp., *Aspergillus*, *Candid*, *Phialosimplex*, *Penicillium*, *Cladosporium*, and *Eutypella* in the lung of COPD patients (Charlson et al., 2012; Su et al., 2015). Thus, bacterial, viral, and fungal infections may lead to exacerbated airway and systemic inflammation (Sethi and Murphy, 2008).

### The Interaction Between Gut and Lung Microbiota in COPD

The research on gut-lung axis in respiratory diseases such as asthma, COPD, cystic fibrosis, and respiratory infections suggested that these diseases might be prevented, or at least ameliorated by regulating gut microbial ecosystem through manipulation of gut microbiota (Zhang D. et al., 2020). The intestinal and respiratory microbiota may affect each other by changing mucosal immunity. On one hand, mice lacking an intestinal microbiota through antibiotic treatment are more susceptible to pneumonia and the alveolar macrophages showed an altered transcriptome which results in decreased phagocytic activity and bacterial clearance (Chen et al., 2011; Schuijt et al., 2016). On the other hand, bacterial and viral respiratory infections in mice lungs lead to gut dysbiosis (Sze et al., 2014; Samuelson et al., 2016). The murine model of sepsis suggested that dysbiosis in gut microbiota influenced composition of the respiratory microbiota through changes in circulating inflammatory cytokines and translocation of intestinal microbiota to the airway (Gauguet et al., 2015).

Clinical data have been available for COPD. The first study that showed increased gastrointestinal (GI) permeability in AECOPD (Sprooten et al., 2018) suggested the involvement of the gut microbiota in exacerbation of COPD. Mucosal epithelial integrity is critical for preventing the entry of harmful particles (e.g., bacteria and their product) into the systemic circulation. Disturbed intestinal integrity may contribute to increased permeability of the intestinal mucosal barrier, resulting in bacterial translocation and systemic inflammation (Turner, 2009). The changes in gut microbiota for COPD have also been observed. For instance, by using untargeted fecal metagenomics and metabolomics, a recent study reported that composition of intestinal microbiota and metabolome in COPD patient was significantly different from those of healthy controls, with an increased abundance of several *Streptococcus parasanguinis_B* in COPD (Bowerman et al., 2020). In an earlier study of AECOPD, it identified increased abundance of both *Streptococcus parasanguinis_B* and *Streptococcus salivarius* in fecal microbiome, while only *Streptococcus parasanguinis_B* was observed increased in sputum (Chan et al., 2015). Furthermore, in a study comparing both gut and lung microbiota in samples collected from 15 AECOPD patients over a 14-day period of antibiotic and steroid treatment, it only observed difference in abundance of certain phyla but not in diversity of core flora between these two sample types (Sun et al., 2020). These studies together suggested a potential cross-talk in the gut-lung axis that may play a critical role in the pathophysiology of COPD, but the mechanisms how the axis regulates microbiota remain unknown.

## Contributing Factors of Dysbiosis in Gut and Lung of COPD

### Cigarette-Smoking Exposure

Cigarette smoking is the most significant risk factor for COPD (Rennard and Vestbo, 2008). Although 15%–20% smokers develop COPD in the United States, approximately 80% COPD patients being past or current smokers (Warren et al., 2014). Smoking is also associated with high mortality rate of COPD (Warren et al., 2014). Although the bacterial communities in bronchoalveolar lavage fluid (BALF) from smokers with COPD were similar to those from healthy smokers and non-smokers, the lung tissues of smokers with COPD showed the heterogeneity and diversity in the lung microbiota at different regions of abnormal lung (Erb-Downward et al., 2011), suggesting that long-term smoking may contribute to the development and exacerbation of COPD. In addition, tobacco smoke has been previously associated with infections of pathogenic microorganisms in the lungs, as well as persistent colonization of opportunistic pathogens in lower respiratory tract, which all in turn lead to exacerbation and progression of COPD (Bagaitkar et al., 2008; Garmendia et al., 2012). Pathophysiologically, this may be established by modification of ciliary movements, damage of tight junctions that increases epithelial permeability, and elevation of neutrophils, macrophages, and T cells along the airways of COPD patients (Garmendia et al., 2012). Notably, exposure to smoke may also modify relative abundance of microbiota of the lungs, as indicated by animal studies (Zhang et al., 2018). Cigarette smoking has been shown to reduce the diversity of microbes in the gut (Faith et al., 2013). However, such smoking-related diversity of gut microbiota has not been compared between COPD patients and healthy smokers.

### Malnutrition

Malnutrition is an important but overlooked problem in COPD (Mete et al., 2018). Around 20% COPD patients have weight loss and malnutrition in protein and calorie, which have negative impact on mortality (Hillas et al., 2015). A 5-year follow-up study showed that individuals that consume less fiber, vitamin, and folic acid are prone to develop airflow limitation and COPD (Jung et al., 2021). The gut microbiome produces a large number of metabolites, e.g., short-chain fatty acids (SCFAs), through fermentation of fiber. SCFAs are potent anti-inflammatory molecules which reduced chemotaxis and adherence in immune cells while increasing the release of anti-inflammatory cytokines and inducing apoptosis (Ratajczak et al., 2019). SCFAs are absorbed into the systemic circulation and can influence lung health. Trompette et al. (2014) showed that dietary fiber leads to altered composition of gut and lung microbiota, and mice with prolonged high-fiber feeding are protected against the allergic inflammation in the lung. A lack of fermentable fibers can lead to malnourishment of the microbiota, resulting in gut dysbiosis and affect lung physiology (Makki et al., 2018; Vaughan et al., 2019).

### Other Factors

Other risk factors such as chronic asthma, low birth weight, childhood respiratory tract infections, pulmonary tuberculosis, and occupational exposures to dusts have also been reported to contribute to the pathogenesis of COPD (Barnes, 2016). The metagenomic analysis of fecal samples from patient with asthma demonstrated a decreased diversity in the gut microbiota compared to a healthy cohort (Wang Q. et al., 2018). In addition, the antibiotics and steroid treatments for COPD seem to have differential effects on lung and gut microbiota (Sun et al., 2020). After treatments with antibiotics and steroid, the abundance and diversity of sputum bacterial flora decreased initially and returned to normal on day 14, whereas the abundance of gut bacteria increased throughout the 14 days post-treatment without affecting diversity of gut microbiota. Of note, these COPD patients have a history of frequent antibiotic use; therefore, the prolonged antibiotic pressure has caused a long-term disturbance in the microbiota with a shift toward bacterial community resistant to antibiotics.

## Molecular Mechanisms Through Gut-Lung Axis in COPD

### Role of NLRP3 Inflammasome Response in COPD

The nucleotide-binding oligomerization domain (NOD)-like receptor (NLR) family pyrin domain-containing protein (NLRP3) inflammasome is generally activated by a range of stimuli and acts as a critical regulator in the innate immunity (Kelley et al., 2019). The NLRP3 inflammasome promotes the recruitment of inflammatory cells and regulates immune response in the gastrointestinal tract and the lung (Donovan et al., 2020). Most bacterial infections in the gut could be cleared *via* gut immunity response. However, for those that escape from the clearance, some has been reported to transfer to circulation and travel to the lung, which results in activation of inflammation. The gut and lung microbiota can influence the function of NLRP3 inflammasome (Donovan et al., 2020). In addition, inflammasomes may also be activated by interactions between bacteria and pattern recognition receptors (PRRs) with alterations of inflammasome responses presented in different severities of COPD (Donovan et al., 2020). Activation of NLRP3 inflammasome mediates caspase-1 activation and the secretion of proinflammatory cytokines such as IL-1b/IL-18. This process was shown to link to the development of airway inflammation in COPD (Wang H. et al., 2018). In peripheral blood mononuclear cells (PBMCs) and bronchial tissues, the mRNA levels of NLRP3, Caspase-1, apoptosis-associated spec-like protein containing CARD (ASC), IL-18, and IL-1b were elevated in AECOPD comparing with smokers; however, the mRNA levels of these gene were decreased in COPD patients in stable stage (Wang H. et al., 2018), suggesting that NLRP3 inflammasome is associated with AECOPD.

Some RCTs have shown that targeting inflammasome-related effectors (i.e., IL-1a and IL-1b) failed to benefit COPD patients at moderate to severe stages. For instance, a 45-week monthly treatment on COPD patients *via* intravenously infusing canakinumab, a human anti-IL-1b monoclonal antibody, did not show statistical difference in forced expiratory volume in 1 s (FEV1) and forced vital capacity (FVC) between treatment and placebo groups (Rogliani et al., 2015). Furthermore, MEDI8968, a human IgG2 monoclonal antibody against IL-1 receptor 1, did not show any beneficial effects on lung function or quality of life of AECOPD patients within 52 weeks of treatment (Calverley et al., 2017). Similar results were obtained for MEDI2338, a human IgG1 monoclonal antibody that binds IL-18, and AZD9056, a P2X7 antagonist that binds human P2X7 receptor. However, some of these RCTs failed to assess the impact of these drugs on disease pathogenesis due to the lack of adequate endpoints; thus, further larger-scaled well-designed studies are required to draw a conclusion for these inflammasome-related effectors.

Immunoproteasome is a specialized type of proteasome required for intracellular protein degradation in immune cells. It also acts as a key regulator for immune cell differentiation, inflammatory activation, and autoimmunity. A recent study demonstrated that proteasome expression was upregulated in PBMCs isolated from severe COPD patients as compared to that from matching controls (Kammerl et al., 2021). The distinct activation of proteasome was observed in PBMCs of young male smokers and severe COPD individuals. The same study also showed that inhibiting immunoproteasome reduced proinflammatory cytokine expression in COPD-derived immune cells (Kammerl et al., 2021), suggesting that targeting immunoproteasome may be a potential treatment for severe COPD.

### Molecular Epigenetic Changes in COPD

Epigenetic modifications have also been identified to participate in altering composition of gut microbiota and in the development of COPD (Zhang L. et al., 2020). However, the exact role of these epigenetic modifications in regulating gut and lung microbiota of COPD remains largely unclear. Firstly, DNA methylation is widely used in the study of epigenetic dysregulation in smokers with or without COPD. DNA methyltransferases (DNMTs) are enzymes catalyzing the addition of a methyl (CH_3_) group to the DNA strands to regulate gene expression. DNMTs are highly sensitive to the availability of nutrients, and they are affected by metabolites produced by gut microbial species (Ansari et al., 2020). DNA methylation is associated with gene expression profile in COPD lung tissues (Lee et al., 2017), however, whether such an epigenetic modification alters COPD-related gut and lung microbiota remains to be further elucidated. Secondly, COPD patients have a reduced level of histone deacetylase 2 (HDAC2) compared with non-smokers (Barnes, 2009), which may results in expansion of inflammation. Other HDACs such as HDAC5, HDAC8, and SIRT1 are all decreased in COPD (Ito et al., 2005; Rajendrasozhan et al., 2008). It is known that gut microbiome can modulate the activity of HDACs by producing SCFAs (Yuille et al., 2018). Therefore, targeting HDAC complex may be a potential therapy for COPD (Zwinderman et al., 2019). Thirdly, increased evidence have shown that gut microbiome plays a key role in regulating microRNAs (miRNAs; Malmuthuge and Guan, 2021), whereas miRNAs are capable of suppressing or preventing COPD development (Zhang L. et al., 2020). Based on profiling analysis, approximately 140 miRNAs are dysregulated in COPD compared to healthy controls, whereas around 70 miRNAs are differentially expressed in COPD compared to smokers (Leidinger et al., 2011; Osei et al., 2015). Clinical data have shown that these miRNA targets were enriched in genes associated with cytokine production (Faiz et al., 2019; Hobbs and Tantisira, 2019). However, the profiling of miRNA has not been compared between lung and gut in COPD. Future studies are required to understand the immune-modulatory role of miRNA in the gut and lung axis.

Epigenetic modifications can be regulated by metabolites of gut microbiota including SCFAs, folates, biotin, and trimethylamine-N-oxide (TMAO). Increased circulating TMAO level was associated with increased mortality of COPD patients, which was independent of disease severity (Ottiger et al., 2018). However, the role of epigenetic modifications on the mechanisms of regulating TMAO levels is still unclear. There are several therapeutic strategies developed to lower TMAO levels to treat atherosclerosis, but with potential side effects (Yang et al., 2019). Whether these therapeutic strategies targeting TMAO levels can be used for COPD should be addressed in future studies. Despite TMAO, the study by Bowerman et al. (2020) also identified a list of metabolites from lipid, amino acids, or xenobiotic classes that are associated with COPD, suggesting these metabolites can be used as biomarkers for COPD diagnosis in the future studies.

## New Therapeutic Approaches for the Management of COPD

### Nutrient-Based Diet

Diet is a readily modifiable factor for COPD patients. A direct association between Western diet and increased risk for COPD has been shown in both genders (Varraso et al., 2015; Mekary, 2016; Young and Hopkins, 2018), indicating that diet is strongly associated with the risk of COPD. For consumptions of specific type of food, several studies have shown that total and cereal dietary fiber are inversely associated with COPD incidence, whereas results of fruits and vegetables are inconsistent (Varraso et al., 2010; Kaluza et al., 2018; Szmidt et al., 2020). Therefore, nutrition-based treatment, particularly high dietary fiber intake, may be a potential non-pharmaceutical approach for managing or preventing COPD.

### Probiotics

A recent study demonstrated that dietary supplementation of probiotics *Lactobacillus rhamnosus* and *Bifidobacteriumbre breve* in COPD mice prevented the airway inflammation and lung damage (Aimbire et al., 2019). The probiotic treatment in these mice attenuated cellularity in BALF and restored the balance between cytokines and chemokines, resulting in a reduction of alveolar enlargement and collagen deposition. Hence, the probiotic treatment yielded positive outcomes in COPD mice, suggesting its potential therapeutic value for the disease, which needs to be testified in large-scaled RCTs for clinical use.

### Fecal Transplantation

Fecal microbial transplantation in humans has shown systemic effects (Li et al., 2014). Dysbiosis in the gut can be modified through fecal transplantation in the cigarette-smoking-based model, indicating that gut microbiota may play a causal role on COPD pathogenesis (Lai et al., 2021). In particular, *Parabacteriodies goldsteinii* (*P. goldsteinii*) was shown to reduce intestinal inflammation, enhance mitochondrial functions, ribosomal activities in colon, restore amino acids metabolism in sera, and inhibit lung infection, at least partially through inhibiting toll-like receptor 4 (TLR4) signaling pathway *via* the lipopolysaccharide derived from *P. goldsteinii* (Lai et al., 2021). Thus, this bacterial strain or its derived lipopolysaccharide could be used as a new therapeutical agent for COPD. At present, no fecal transplantation studies have been conducted in COPD patients, hence, whether this approach is effective for COPD awaits further clinical investigations.

## Discussion

COPD is a common but poorly recognized disease worldwide (Agusti et al., 2020). Current management strategies for COPD have focused on alleviating symptoms and preventing exacerbation. However, new interventions are required to improve predictiveness, preventiveness, and personalization. While recent research highlights the significance of the gut-lung axis in COPD, there is still limited laboratory data for its related mechanism and clinical data for its practical use. We need to put more efforts to clarify how gut microbiota is affected in COPD and how gut dysbiosis can be modified to treat the disease. In addition to exploring the change in diversity and relative abundance of gut microbiota, we should also concentrate on investigating the underlying mechanisms to better understand whether a particular interventional approach exerts beneficial and protective effects on diseased individuals and how it works. For instance, the modulation of innate immunity and systemic inflammation (as indicated by specific inflammatory markers), maintenance of epithelial integrity from microbial invasion, and stimulation of beneficial bacteria that generate specific metabolites [e.g., small chain fatty acids (SCFAs)]. Furthermore, multicenter analysis could be considered as different demographic backgrounds may contribute to the diversity of microbiota in the gut and lung. Lastly, longitudinal studies are crucial, as they may represent a change, particularly disease-related change of an individual, which may assist with revealing the role gut-lung axis played in COPD.

It is noteworthy that region of sampling may affect evaluating microbiota dysbiosis in the gut-lung axis. For instance, though considered sterile in the past, the lower respiratory tract has now been considered to host smaller amount of the microbiota. For gut microbiota, although fecal specimens are more commonly used due to easy accessibility, the colonized germs vary significantly in different regions of gastrointestinal tract. Therefore, sampling methods should be carefully determined in the design of future studies.

Several risk factors, including smoking, diet, antibiotics, and steroid treatments, are involved in affecting the gut-lung axis. The cross-talk between the gut and the lung has been hypothesized to involve various pathways, probably *via* NLRP3 inflammasome, COPD-related epigenetic modification, immunological changes, and various microbiota-specific metabolites. However, the significance and mechanism of each factor in regulating the gut-lung axis remain unclear yet. In addition, previous studies have highlighted the potential use of metabolites, miRNAs, or miRNA targets to be used as diagnostic biomarkers. Therefore, further investigation will help to determine whether these are cost-effective ways for COPD diagnosis. The relatively successful modification by using antibiotics, probiotics, and prebiotics add evidence of the crucial part microbiota dysbiosis played in the gut-lung axis.

Gut dysbiosis in COPD may be a modifiable factor to be developed as a non-drug therapeutical strategy for the prevention and management of the disease, as studies of nutrient-based diet have shown positive results in reducing COPD risk. Meanwhile, probiotic supplementation and fecal transplantation have also shown positive outcomes in murine COPD models. More RCTs are required to investigate the effectiveness of these interventions on the prevention and treatment of COPD.

In summary, the gut-lung axis is highly associated with COPD and the gut and lung microbiota can influence lung immunity and health. Modifying the gut microbiota could be an effective strategy to treat or even prevent COPD. Further studies are required to understand the role of gut-lung axis in the pathogenies of COPD and lung health.

## Author Contributions

LQ, QC, and YZ performed literature search, drafted the manuscript, and prepared the figures. YW and HM edited and revised the manuscript. YZ initiated, supervised, and finalized the work for submission. All authors contributed to the article and approved the submitted version.

## Funding

This work was supported by the Scientific and Technological Innovation Foundation of Health Commission of Putuo District of China (ptkwws202118); the Improvement Project of Specialized Diseases of Putuo District of China (2020tszb05).

## Conflict of Interest

The authors declare that the research was conducted in the absence of any commercial or financial relationships that could be construed as a potential conflict of interest.

## Publisher’s Note

All claims expressed in this article are solely those of the authors and do not necessarily represent those of their affiliated organizations, or those of the publisher, the editors and the reviewers. Any product that may be evaluated in this article, or claim that may be made by its manufacturer, is not guaranteed or endorsed by the publisher.

## References

[ref1] Agusti A. Vogelmeier C. Faner R. (2020). COPD 2020: changes and challenges. Am. J. Phys. Lung Cell. Mol. Phys. 319, L879–L883. doi: 10.1152/ajplung.00429.2020, PMID: 32964724

[ref2] Aimbire F. Carvalho J. L. Fialho A. K. Miranda M. Albertini R. Keller A. (2019). Role of probiotics *Bfidobacterium breve* and *Lactobacillus rhmanosus* on lung inflammation and airway remodeling in an experimental model of chronic obstructive pulmonary disease. Eur. Respir. J. 54:PA2452. doi: 10.1183/13993003.congress-2019.PA2452

[ref3] Al Bander Z. Nitert M. D. Mousa A. Naderpoor N. (2020). The gut microbiota and inflammation: an overview. Int. J. Environ. Res. Public Health 17:7618. doi: 10.3390/ijerph17207618, PMID: 33086688 PMC7589951

[ref4] Ansari I. Raddatz G. Gutekunst J. Ridnik M. Cohen D. Abu-Remaileh M.. (2020). The microbiota programs DNA methylation to control intestinal homeostasis and inflammation. Nat. Microbiol. 5, 610–619. doi: 10.1038/s41564-019-0659-3, PMID: 32015497

[ref5] Bagaitkar J. Demuth D. R. Scott D. A. (2008). Tobacco use increases susceptibility to bacterial infection. Tob. Induc. Dis. 4:12. doi: 10.1186/1617-9625-4-12, PMID: 19094204 PMC2628337

[ref6] Barnes P. J. (2009). Role of HDAC2 in the pathophysiology of COPD. Annu. Rev. Physiol. 71, 451–464. doi: 10.1146/annurev.physiol.010908.163257, PMID: 18817512

[ref7] Barnes P. J. (2016). Inflammatory mechanisms in patients with chronic obstructive pulmonary disease. J. Allergy Clin. Immunol. 138, 16–27. doi: 10.1016/j.jaci.2016.05.011 27373322

[ref8] Bowerman K. L. Rehman S. F. Vaughan A. Lachner N. Budden K. F. Kim R. Y.. (2020). Disease-associated gut microbiome and metabolome changes in patients with chronic obstructive pulmonary disease. Nat. Commun. 11:5886. doi: 10.1038/s41467-020-19701-0, PMID: 33208745 PMC7676259

[ref9] Budden K. F. Gellatly S. L. Wood D. L. Cooper M. A. Morrison M. Hugenholtz P.. (2017). Emerging pathogenic links between microbiota and the gut-lung axis. Nat. Rev. Microbiol. 15, 55–63. doi: 10.1038/nrmicro.2016.142, PMID: 27694885

[ref10] Budden K. F. Shukla S. D. Rehman S. F. Bowerman K. L. Keely S. Hugenholtz P.. (2019). Functional effects of the microbiota in chronic respiratory disease. Lancet Respir. Med. 7, 907–920. doi: 10.1016/S2213-2600(18)30510-1, PMID: 30975495

[ref11] Calverley P. M. A. Sethi S. Dawson M. Ward C. K. Finch D. K. Penney M.. (2017). A randomised, placebo-controlled trial of anti-interleukin-1 receptor 1 monoclonal antibody MEDI8968 in chronic obstructive pulmonary disease. Respir. Res. 18:153. doi: 10.1186/s12931-017-0633-7, PMID: 28793896 PMC5551010

[ref12] Chan K. G. Ng K. T. Pang Y. K. Chong T. M. Kamarulzaman A. Yin W. F.. (2015). Genome anatomy of *Streptococcus parasanguinis* strain C1A, isolated from a patient with acute exacerbation of chronic obstructive pulmonary disease, reveals unusual genomic features. Genome Announc. 3, e00541–e00615. doi: 10.1128/genomeA.00541-15, PMID: 26021924 PMC4447909

[ref13] Charlson E. S. Diamond J. M. Bittinger K. Fitzgerald A. S. Yadav A. Haas A. R.. (2012). Lung-enriched organisms and aberrant bacterial and fungal respiratory microbiota after lung transplant. Am. J. Respir. Crit. Care Med. 186, 536–545. doi: 10.1164/rccm.201204-0693OC, PMID: 22798321 PMC3480531

[ref14] Chen L. W. Chen P. H. Hsu C. M. (2011). Commensal microflora contribute to host defense against *Escherichia coli* pneumonia through toll-like receptors. Shock 36, 67–75. doi: 10.1097/SHK.0b013e3182184ee7, PMID: 21412185

[ref15] Chotirmall S. H. Gellatly S. L. Budden K. F. Mac Aogain M. Shukla S. D. Wood D. L.. (2017). Microbiomes in respiratory health and disease: an Asia-Pacific perspective. Respirology 22, 240–250. doi: 10.1111/resp.12971, PMID: 28102970

[ref16] Chunxi L. Haiyue L. Yanxia L. Jianbing P. Jin S. (2020). The gut microbiota and respiratory diseases: new evidence. J. Immunol. Res. 2020:2340670. doi: 10.1155/2020/2340670, PMID: 32802893 PMC7415116

[ref17] Criner G. J. Dreher M. D'Ambrosio C. M. Zuwallack R. Geiseler J. Pepin J. L. (2018). COPD advanced patient management. Chest 153, 1497–1498. doi: 10.1016/j.chest.2018.03.054 29884254

[ref18] Dicker A. J. Crichton M. L. Cassidy A. J. Brady G. Hapca A. Tavendale R.. (2018). Genetic mannose binding lectin deficiency is associated with airway microbiota diversity and reduced exacerbation frequency in COPD. Thorax 73, 510–518. doi: 10.1136/thoraxjnl-2016-209931, PMID: 29101284 PMC5969339

[ref19] Donovan C. Liu G. Shen S. Marshall J. E. Kim R. Y. Alemao C. A.. (2020). The role of the microbiome and the NLRP3 inflammasome in the gut and lung. J. Leukoc. Biol. 108, 925–935. doi: 10.1002/JLB.3MR0720-472RR, PMID: 33405294

[ref20] Erb-Downward J. R. Thompson D. L. Han M. K. Freeman C. M. McCloskey L. Schmidt L. A.. (2011). Analysis of the lung microbiome in the "healthy" smoker and in COPD. PLoS One 6:e16384. doi: 10.1371/journal.pone.0016384, PMID: 21364979 PMC3043049

[ref21] Faith J. J. Guruge J. L. Charbonneau M. Subramanian S. Seedorf H. Goodman A. L.. (2013). The long-term stability of the human gut microbiota. Science 341:1237439. doi: 10.1126/science.1237439, PMID: 23828941 PMC3791589

[ref22] Faiz A. Steiling K. Roffel M. P. Postma D. S. Spira A. Lenburg M. E.. (2019). Effect of long-term corticosteroid treatment on microRNA and gene-expression profiles in COPD. Eur. Respir. J. 53:1801202, 1801202. doi: 10.1183/13993003.01202-2018, PMID: 30846474

[ref23] Garcia-Nunez M. Millares L. Pomares X. Ferrari R. Perez-Brocal V. Gallego M.. (2014). Severity-related changes of bronchial microbiome in chronic obstructive pulmonary disease. J. Clin. Microbiol. 52, 4217–4223. doi: 10.1128/JCM.01967-14, PMID: 25253795 PMC4313290

[ref24] Garmendia J. Morey P. Bengoechea J. A. (2012). Impact of cigarette smoke exposure on host-bacterial pathogen interactions. Eur. Respir. J. 39, 467–477. doi: 10.1183/09031936.00061911, PMID: 21737564

[ref25] Gauguet S. D'Ortona S. Ahnger-Pier K. Duan B. Surana N. K. Lu R.. (2015). Intestinal microbiota of mice influences resistance to *Staphylococcus aureus* pneumonia. Infect. Immun. 83, 4003–4014. doi: 10.1128/IAI.00037-15, PMID: 26216419 PMC4567647

[ref26] Girosi D. Bellodi S. Sabatini F. Rossi G. A. (2006). The lung and the gut: common origins, close links. Paediatr. Respir. Rev. 7(Suppl 1), S235–S239. doi: 10.1016/j.prrv.2006.04.192, PMID: 16798577

[ref27] Hillas G. Perlikos F. Tsiligianni I. Tzanakis N. (2015). Managing comorbidities in COPD. Int. J. Chronic Obstr. Pulm. Dis. 10, 95–109. doi: 10.2147/COPD.S54473, PMID: 25609943 PMC4293292

[ref28] Hobbs B. D. Tantisira K. G. (2019). MicroRNAs in COPD: small molecules with big potential. Eur. Respir. J. 53:1900515. doi: 10.1183/13993003.00515-2019, PMID: 31023868

[ref29] Huang Y. J. Sethi S. Murphy T. Nariya S. Boushey H. A. Lynch S. V. (2014). Airway microbiome dynamics in exacerbations of chronic obstructive pulmonary disease. J. Clin. Microbiol. 52, 2813–2823. doi: 10.1128/JCM.00035-14, PMID: 24850358 PMC4136157

[ref30] Ito K. Ito M. Elliott W. M. Cosio B. Caramori G. Kon O. M.. (2005). Decreased histone deacetylase activity in chronic obstructive pulmonary disease. N. Engl. J. Med. 352, 1967–1976. doi: 10.1056/NEJMoa041892 15888697

[ref31] Jandhyala S. M. Talukdar R. Subramanyam C. Vuyyuru H. Sasikala M. Nageshwar Reddy D. (2015). Role of the normal gut microbiota. World J. Gastroenterol. 21, 8787–8803. doi: 10.3748/wjg.v21.i29.8787, PMID: 26269668 PMC4528021

[ref32] Jiang T. T. Shao T. Y. Ang W. X. G. Kinder J. M. Turner L. H. Pham G.. (2017). Commensal fungi recapitulate the protective benefits of intestinal bacteria. Cell Host Microbe 22, 809.e4–816.e4. doi: 10.1016/j.chom.2017.10.013, PMID: 29174402 PMC5730478

[ref33] Jung Y. J. Lee S. H. Chang J. H. Lee H. S. Kang E. H. Lee S. W. (2021). The impact of changes in the intake of fiber and antioxidants on the development of chronic obstructive pulmonary disease. Nutrients 13:580. doi: 10.3390/nu13020580, PMID: 33578669 PMC7916350

[ref34] Kaluza J. Harris H. Wallin A. Linden A. Wolk A. (2018). Dietary fiber intake and risk of chronic obstructive pulmonary disease: a prospective cohort study of men. Epidemiology 29, 254–260. doi: 10.1097/EDE.0000000000000750, PMID: 28901975

[ref35] Kammerl I. E. Hardy S. Flexeder C. Urmann A. Peierl J. Wang Y.. (2021). Activation of immune cell proteasomes in peripheral blood of smokers and COPD patients: implications for therapy. Eur. Respir. J. 59:2101798. doi: 10.1183/13993003.01798-2021, PMID: 34561290 PMC8891681

[ref36] Kelley N. Jeltema D. Duan Y. He Y. (2019). The NLRP3 Inflammasome: an overview of mechanisms of activation and regulation. Int. J. Mol. Sci. 20:3328. doi: 10.3390/ijms20133328, PMID: 31284572 PMC6651423

[ref37] Lai H. C. Lin T. L. Chen T. W. Kuo Y. L. Chang C. J. Wu T. R.. (2021). Gut microbiota modulates COPD pathogenesis: role of anti-inflammatory *Parabacteroides goldsteinii* lipopolysaccharide. Gut 71, 309–321. doi: 10.1136/gutjnl-2020-322599, PMID: 33687943

[ref38] Lee M. K. Hong Y. Kim S. Y. Kim W. J. London S. J. (2017). Epigenome-wide association study of chronic obstructive pulmonary disease and lung function in Koreans. Epigenomics 9, 971–984. doi: 10.2217/epi-2017-0002, PMID: 28621160 PMC5674213

[ref39] Leidinger P. Keller A. Borries A. Huwer H. Rohling M. Huebers J.. (2011). Specific peripheral miRNA profiles for distinguishing lung cancer from COPD. Lung Cancer 74, 41–47. doi: 10.1016/j.lungcan.2011.02.003, PMID: 21388703

[ref40] Li Q. Wang C. Tang C. He Q. Zhao X. Li N.. (2014). Therapeutic modulation and reestablishment of the intestinal microbiota with fecal microbiota transplantation resolves sepsis and diarrhea in a patient. Am. J. Gastroenterol. 109, 1832–1834. doi: 10.1038/ajg.2014.299 25373588

[ref41] Limon J. J. Skalski J. H. Underhill D. M. (2017). Commensal fungi in health and disease. Cell Host Microbe 22, 156–165. doi: 10.1016/j.chom.2017.07.002, PMID: 28799901 PMC5573128

[ref42] Makki K. Deehan E. C. Walter J. Backhed F. (2018). The impact of dietary fiber on gut microbiota in host health and disease. Cell Host Microbe 23, 705–715. doi: 10.1016/j.chom.2018.05.012 29902436

[ref43] Mallia P. Message S. D. Gielen V. Contoli M. Gray K. Kebadze T.. (2011). Experimental rhinovirus infection as a human model of chronic obstructive pulmonary disease exacerbation. Am. J. Respir. Crit. Care Med. 183, 734–742. doi: 10.1164/rccm.201006-0833OC, PMID: 20889904 PMC3081284

[ref44] Malmuthuge N. Guan L. L. (2021). Noncoding RNAs: regulatory molecules of host-microbiome crosstalk. Trends Microbiol. 29, 713–724. doi: 10.1016/j.tim.2020.12.003, PMID: 33419590

[ref45] Mateer S. W. Maltby S. Marks E. Foster P. S. Horvat J. C. Hansbro P. M.. (2015). Potential mechanisms regulating pulmonary pathology in inflammatory bowel disease. J. Leukoc. Biol. 98, 727–737. doi: 10.1189/jlb.3RU1114-563R, PMID: 26307547

[ref46] Mateer S. W. Mathe A. Bruce J. Liu G. Maltby S. Fricker M.. (2018). IL-6 drives neutrophil-mediated pulmonary inflammation associated with bacteremia in murine models of colitis. Am. J. Pathol. 188, 1625–1639. doi: 10.1016/j.ajpath.2018.03.016, PMID: 29684360

[ref47] Mayhew D. Devos N. Lambert C. Brown J. R. Clarke S. C. Kim V. L.. (2018). Longitudinal profiling of the lung microbiome in the AERIS study demonstrates repeatability of bacterial and eosinophilic COPD exacerbations. Thorax 73, 422–430. doi: 10.1136/thoraxjnl-2017-210408, PMID: 29386298 PMC5909767

[ref48] Mekary R. A. (2016). A higher overall diet quality is inversely associated with the risk of chronic obstructive pulmonary disease (COPD) in men and women. Evid. Based Med. 21:36. doi: 10.1136/ebmed-2015-110193, PMID: 26486416

[ref49] Mete B. Pehlivan E. Gulbas G. Gunen H. (2018). Prevalence of malnutrition in COPD and its relationship with the parameters related to disease severity. Int. J. Chronic Obstr. Pulm. Dis. 13, 3307–3312. doi: 10.2147/COPD.S179609, PMID: 30349235 PMC6188194

[ref50] Millares L. Ferrari R. Gallego M. Garcia-Nunez M. Perez-Brocal V. Espasa M.. (2014). Bronchial microbiome of severe COPD patients colonised by *Pseudomonas aeruginosa*. Eur. J. Clin. Microbiol. Infect. Dis. 33, 1101–1111. doi: 10.1007/s10096-013-2044-0, PMID: 24449346 PMC4042013

[ref51] Mjosberg J. Rao A. (2018). Lung inflammation originating in the gut. Science 359, 36–37. doi: 10.1126/science.aar4301, PMID: 29302003

[ref52] Nash A. K. Auchtung T. A. Wong M. C. Smith D. P. Gesell J. R. Ross M. C.. (2017). The gut mycobiome of the human microbiome project healthy cohort. Microbiome 5:153. doi: 10.1186/s40168-017-0373-4, PMID: 29178920 PMC5702186

[ref53] Osei E. T. Florez-Sampedro L. Timens W. Postma D. S. Heijink I. H. Brandsma C. A. (2015). Unravelling the complexity of COPD by microRNAs: it's a small world after all. Eur. Respir. J. 46, 807–818. doi: 10.1183/13993003.02139-2014, PMID: 26250493

[ref54] Ottiger M. Nickler M. Steuer C. Bernasconi L. Huber A. Christ-Crain M.. (2018). Gut, microbiota-dependent trimethylamine-N-oxide is associated with long-term all-cause mortality in patients with exacerbated chronic obstructive pulmonary disease. Nutrition 45, 135.e1–141.e1. doi: 10.1016/j.nut.2017.07.001, PMID: 28870405

[ref55] Qin J. Li R. Raes J. Arumugam M. Burgdorf K. S. Manichanh C.. (2010). A human gut microbial gene catalogue established by metagenomic sequencing. Nature 464, 59–65. doi: 10.1038/nature08821, PMID: 20203603 PMC3779803

[ref56] Raherison C. Girodet P. O. (2009). Epidemiology of COPD. Eur. Respir. Rev. 18, 213–221. doi: 10.1183/09059180.00003609 20956146

[ref57] Rajendrasozhan S. Yang S. R. Kinnula V. L. Rahman I. (2008). SIRT1, an antiinflammatory and antiaging protein, is decreased in lungs of patients with chronic obstructive pulmonary disease. Am. J. Respir. Crit. Care Med. 177, 861–870. doi: 10.1164/rccm.200708-1269OC, PMID: 18174544 PMC2292827

[ref58] Ratajczak W. Ryl A. Mizerski A. Walczakiewicz K. Sipak O. Laszczynska M. (2019). Immunomodulatory potential of gut microbiome-derived short-chain fatty acids (SCFAs). Acta Biochim. Pol. 66, 1–12. doi: 10.18388/abp.2018_2648, PMID: 30831575

[ref59] Rennard S. I. Vestbo J. (2008). Natural histories of chronic obstructive pulmonary disease. Proc. Am. Thorac. Soc. 5, 878–883. doi: 10.1513/pats.200804-035QC, PMID: 19056710 PMC2720106

[ref60] Rogliani P. Calzetta L. Ora J. Matera M. G. (2015). Canakinumab for the treatment of chronic obstructive pulmonary disease. Pulm. Pharmacol. Ther. 31, 15–27. doi: 10.1016/j.pupt.2015.01.005, PMID: 25660162

[ref61] Rosell A. Monso E. Soler N. Torres F. Angrill J. Riise G.. (2005). Microbiologic determinants of exacerbation in chronic obstructive pulmonary disease. Arch. Intern. Med. 165, 891–897. doi: 10.1001/archinte.165.8.891, PMID: 15851640

[ref62] Samuelson D. R. Charles T. P. de la Rua N. M. Taylor C. M. Blanchard E. E. Luo M.. (2016). Analysis of the intestinal microbial community and inferred functional capacities during the host response to pneumocystis pneumonia. Exp. Lung Res. 42, 425–439. doi: 10.1080/01902148.2016.1258442, PMID: 27925857 PMC5304582

[ref63] Schols A. M. Ferreira I. M. Franssen F. M. Gosker H. R. Janssens W. Muscaritoli M.. (2014). Nutritional assessment and therapy in COPD: a European Respiratory Society statement. Eur. Respir. J. 44, 1504–1520. doi: 10.1183/09031936.00070914, PMID: 25234804

[ref64] Schuijt T. J. Lankelma J. M. Scicluna B. P. de Sousa e Melo F. Roelofs J. J. de Boer J. D.. (2016). The gut microbiota plays a protective role in the host defence against pneumococcal pneumonia. Gut 65, 575–583. doi: 10.1136/gutjnl-2015-309728, PMID: 26511795 PMC4819612

[ref65] Sender R. Fuchs S. Milo R. (2016). Revised estimates for the number of human and bacteria cells in the body. PLoS Biol. 14:e1002533. doi: 10.1371/journal.pbio.1002533, PMID: 27541692 PMC4991899

[ref66] Sethi S. Murphy T. F. (2008). Infection in the pathogenesis and course of chronic obstructive pulmonary disease. N. Engl. J. Med. 359, 2355–2365. doi: 10.1056/NEJMra0800353 19038881

[ref67] Shukla S. D. Budden K. F. Neal R. Hansbro P. M. (2017). Microbiome effects on immunity, health and disease in the lung. Clin. Transl. Immunol. 6:e133. doi: 10.1038/cti.2017.6, PMID: 28435675 PMC5382435

[ref68] Sprooten R. T. M. Lenaerts K. Braeken D. C. W. Grimbergen I. Rutten E. P. Wouters E. F. M.. (2018). Increased small intestinal permeability during severe acute exacerbations of COPD. Respiration 95, 334–342. doi: 10.1159/000485935, PMID: 29393240 PMC5985742

[ref69] Su J. Liu H. Y. Tan X. L. Ji Y. Jiang Y. X. Prabhakar M.. (2015). Sputum bacterial and fungal dynamics during exacerbations of severe COPD. PLoS One 10:e0130736. doi: 10.1371/journal.pone.0130736, PMID: 26147303 PMC4493005

[ref70] Sun Z. Zhu Q. L. Shen Y. Yan T. Zhou X. (2020). Dynamic changes of gut and lung microorganisms during chronic obstructive pulmonary disease exacerbations. Kaohsiung J. Med. Sci. 36, 107–113. doi: 10.1002/kjm2.12147, PMID: 31782610 PMC11896544

[ref71] Sze M. A. Tsuruta M. Yang S. W. Oh Y. Man S. F. Hogg J. C.. (2014). Changes in the bacterial microbiota in gut, blood, and lungs following acute LPS instillation into mice lungs. PLoS One 9:e111228. doi: 10.1371/journal.pone.0111228, PMID: 25333938 PMC4205020

[ref72] Szmidt M. K. Kaluza J. Harris H. R. Linden A. Wolk A. (2020). Long-term dietary fiber intake and risk of chronic obstructive pulmonary disease: a prospective cohort study of women. Eur. J. Nutr. 59, 1869–1879. doi: 10.1007/s00394-019-02038-w, PMID: 31280344 PMC7351821

[ref73] Tan D. B. Amran F. S. Teo T. H. Price P. Moodley Y. P. (2016). Levels of CMV-reactive antibodies correlate with the induction of CD28(null) T cells and systemic inflammation in chronic obstructive pulmonary disease (COPD). Cell. Mol. Immunol. 13, 551–553. doi: 10.1038/cmi.2015.4, PMID: 27402584 PMC4947821

[ref74] Trompette A. Gollwitzer E. S. Yadava K. Sichelstiel A. K. Sprenger N. Ngom-Bru C.. (2014). Gut microbiota metabolism of dietary fiber influences allergic airway disease and hematopoiesis. Nat. Med. 20, 159–166. doi: 10.1038/nm.3444, PMID: 24390308

[ref75] Tso G. H. W. Reales-Calderon J. A. Tan A. S. M. Sem X. Le G. T. T. Tan T. G.. (2018). Experimental evolution of a fungal pathogen into a gut symbiont. Science 362, 589–595. doi: 10.1126/science.aat0537, PMID: 30385579

[ref76] Turner J. R. (2009). Intestinal mucosal barrier function in health and disease. Nat. Rev. Immunol. 9, 799–809. doi: 10.1038/nri2653 19855405

[ref77] Utokaparch S. Sze M. A. Gosselink J. V. McDonough J. E. Elliott W. M. Hogg J. C.. (2014). Respiratory viral detection and small airway inflammation in lung tissue of patients with stable, mild COPD. J. Chronic Obstr. Pulm. Dis. 11, 197–203. doi: 10.3109/15412555.2013.836166 24088037

[ref78] Varraso R. Chiuve S. E. Fung T. T. Barr R. G. Hu F. B. Willett W. C.. (2015). Alternate healthy eating index 2010 and risk of chronic obstructive pulmonary disease among US women and men: prospective study. BMJ 350:h286. doi: 10.1136/bmj.h286, PMID: 25649042 PMC4707519

[ref79] Varraso R. Willett W. C. Camargo C. A. Jr. (2010). Prospective study of dietary fiber and risk of chronic obstructive pulmonary disease among US women and men. Am. J. Epidemiol. 171, 776–784. doi: 10.1093/aje/kwp455, PMID: 20172921 PMC2877480

[ref80] Vaughan A. Frazer Z. A. Hansbro P. M. Yang I. A. (2019). COPD and the gut-lung axis: the therapeutic potential of fibre. J. Thorac. Dis. 11, S2173–S2180. doi: 10.21037/jtd.2019.10.40, PMID: 31737344 PMC6831926

[ref81] Vestbo J. Hurd S. S. Agusti A. G. Jones P. W. Vogelmeier C. Anzueto A.. (2013). Global strategy for the diagnosis, management, and prevention of chronic obstructive pulmonary disease: GOLD executive summary. Am. J. Respir. Crit. Care Med. 187, 347–365. doi: 10.1164/rccm.201204-0596PP 22878278

[ref82] Wang Z. Bafadhel M. Haldar K. Spivak A. Mayhew D. Miller B. E.. (2016). Lung microbiome dynamics in COPD exacerbations. Eur. Respir. J. 47, 1082–1092. doi: 10.1183/13993003.01406-2015, PMID: 26917613

[ref83] Wang Q. Li F. Liang B. Liang Y. Chen S. Mo X.. (2018). A metagenome-wide association study of gut microbiota in asthma in UK adults. BMC Microbiol. 18:114. doi: 10.1186/s12866-018-1257-x, PMID: 30208875 PMC6134768

[ref84] Wang H. Lv C. Wang S. Ying H. Weng Y. Yu W. (2018). NLRP3 inflammasome involves in the acute exacerbation of patients with chronic obstructive pulmonary disease. Inflammation 41, 1321–1333. doi: 10.1007/s10753-018-0780-0, PMID: 29656319

[ref85] Wang Z. Singh R. Miller B. E. Tal-Singer R. Van Horn S. Tomsho L.. (2018). Sputum microbiome temporal variability and dysbiosis in chronic obstructive pulmonary disease exacerbations: an analysis of the COPDMAP study. Thorax 73, 331–338. doi: 10.1136/thoraxjnl-2017-210741, PMID: 29269441

[ref86] Warren G. W. Alberg A. J. Kraft A. S. Cummings K. M. (2014). The 2014 surgeon General's report: "The health consequences of smoking-50 years of progress": a paradigm shift in cancer care. Cancer 120, 1914–1916. doi: 10.1002/cncr.28695, PMID: 24687615 PMC5928784

[ref87] Wedzicha J. A. Seemungal T. A. (2007). COPD exacerbations: defining their cause and prevention. Lancet 370, 786–796. doi: 10.1016/S0140-6736(07)61382-8, PMID: 17765528 PMC7134993

[ref88] Yang S. Li X. Yang F. Zhao R. Pan X. Liang J.. (2019). Gut microbiota-dependent marker TMAO in promoting cardiovascular disease: inflammation mechanism, clinical prognostic, and potential as a therapeutic target. Front. Pharmacol. 10:1360. doi: 10.3389/fphar.2019.01360, PMID: 31803054 PMC6877687

[ref89] Young R. P. Hopkins R. J. (2018). Is the “Western diet” a new smoking gun for chronic obstructive pulmonary disease? Ann. Am. Thorac. Soc. 15, 662–663. doi: 10.1513/AnnalsATS.201802-131ED, PMID: 29856249

[ref90] Yuille S. Reichardt N. Panda S. Dunbar H. Mulder I. E. (2018). Human gut bacteria as potent class I histone deacetylase inhibitors in vitro through production of butyric acid and valeric acid. PLoS One 13:e0201073. doi: 10.1371/journal.pone.0201073, PMID: 30052654 PMC6063406

[ref91] Zhang R. Chen L. Cao L. Li K. J. Huang Y. Luan X. Q.. (2018). Effects of smoking on the lower respiratory tract microbiome in mice. Respir. Res. 19:253. doi: 10.1186/s12931-018-0959-9, PMID: 30547792 PMC6295055

[ref92] Zhang D. Li S. Wang N. Tan H. Y. Zhang Z. Feng Y. (2020). The cross-talk between gut microbiota and lungs in common lung diseases. Front. Microbiol. 11:301. doi: 10.3389/fmicb.2020.00301, PMID: 32158441 PMC7052046

[ref93] Zhang L. Valizadeh H. Alipourfard I. Bidares R. Aghebati-Maleki L. Ahmadi M. (2020). Epigenetic modifications and therapy in chronic obstructive pulmonary disease (COPD): an update review. J. Chronic Obstr. Pulm. Dis. 17, 333–342. doi: 10.1080/15412555.2020.1780576, PMID: 32558592

[ref94] Zwinderman M. R. H. de Weerd S. Dekker F. J. (2019). Targeting HDAC complexes in asthma and COPD. Epigenomes 3:19. doi: 10.3390/epigenomes3030019, PMID: 34968229 PMC8594684

